# Microbiological evaluation in oral health units: detection of antibiotic resistant bacteria

**DOI:** 10.1080/07853890.2021.1895586

**Published:** 2021-09-28

**Authors:** Inês Gama, Helena Barroso

**Affiliations:** a Instituto Universitário Egas Moniz (IUEM), Caparica, Portugal; b Centro de Investigação Interdisciplinar Egas Moniz (CiiEM), IUEM; Cooperativa de Ensino Superior, C.R.L., Caparica, Portugal

## Abstract

**Introduction:**

The environment in oral health care units can represent an important source of transmission of infections, which can be acquired through aerosols, bleeding, saliva and respiratory secretions [1]. It is increasingly important to prevent cross-infection in dental clinics [2]. Resistance to antibiotics is a serious public health problem. Presence of resistant microorganisms in health care units is a worrying reality, but little is known about oral health care units [3]. The aim of this study was the detection of microorganisms resistant to antibiotics at the Clínica Dentária Egas Moniz, in the dentist’s chair, trays and lamp handles.

**Materials and methods:**

Environmental samples were collected at the dental clinic with a swab. Sampling was made at trays, chairs and lamp handles, at the end of the appointments. All samples were inoculated in Trypticase soy agar (TSA), Mannitol salt agar (MSA) and MacConkey agar. All the bacteria that grew in MSA and were mannitol positive were inoculated in chromogenic agar, because we wanted to detect Methicillin resistant *Staphylococcus aureus* (MRSA).

**Results:**

Of the 123 samples obtained in 41 working stations, only two (1.6%) lactose negative bacteria were found. One was isolated from a tray and the other from a lamp handle, in two different working stations. We found 51 mannitol positive *Staphylococcus* samples (41.5%), were isolated from 36 different working stations, being 14 samples identified as MRSA (11.4%). These MRSA were isolated from 13 different working stations. In our study, we cannot identify if there was a preferential location for the presence of MRSA, but we found it mainly at trays and dentist’s chairs.

**Discussion and conclusions:**

There was a low contamination by *Enterobacteria*ceae. However, a percentage of MRSA isolation of 11.4% was obtained. There are few similar studies. Although, in a study where 95 surfaces from 7 different university dental clinics were evaluated, 8 MRSA were found, which corresponds to 8.4%. Comparing to our study, we obtained a slightly higher percentage. These results demonstrate that oral health care units are also sites of contamination, where bacteria with resistance to antibiotics can circulate. They also reinforce the need for good hygiene and disinfection of the site between appointments.
